# Imported cases of cutaneous leishmaniasis in Cuba, 2017: role of human movement

**DOI:** 10.1186/s40794-022-00171-9

**Published:** 2022-07-01

**Authors:** Lianet Monzote, Daniel González, Orestes Blanco, Jorge Fraga, Virginia Capó, Alberto Herrera, Ana Margarita Montalvo

**Affiliations:** 1 Parasitology Department, Instituto de Medicina Tropical Pedro Kourí, Autopista Novia del Mediodía Km 6 1/2, La Habana, Cuba; 2 Department of Medicine, Instituto de Medicina Tropical Pedro Kourí, Autopista Novia del Mediodía Km 6 1/2, La Habana, Cuba; 3 Department of Science and Innovation, Instituto de Medicina Tropical Pedro Kourí, Autopista Novia del Mediodía Km 6 1/2, La Habana, Cuba; 4 Pathology Department, Instituto de Medicina Tropical Pedro Kourí, Autopista Novia del Mediodía Km 6 1/2, La Habana, Cuba; 5 Former researcher of Instituto de Medicina Tropical Pedro Kourí, (Head of Leishmania Group until 2019), Autopista Novia del Mediodía Km 6 1/2, La Habana, Cuba

## Abstract

**Background:**

Leishmaniasis is a vector-borne disease caused by several species from genus *Leishmania*. An increase in the number of cases related to human movement has been informed in the last years. Due to the increase of suspicious leishmaniasis cases arriving in Cuba during 2017, a general analysis is presented herein.

**Methods:**

Clinical samples were collected from 5 patients suspicious of leishmaniasis, received from January to December 2017 at the Institute of Tropical Medicine Pedro Kourí, Cuba. Skin lesion samples were analyzed using different diagnostic assays: direct smear, histological examination, and molecular analysis for species identification. Epidemiological and demographic data were requested from each case and analyzed. Treatment and follow up of patient was also performed.

**Results:**

Five cases were confirmed as *Leishmania* infection according to microscopic observation and molecular methods results. PCR-18S, PCR-N/RFLP and PCR-F/RFLP identified the following species: *L. panamensis* (2 cases), *L. braziliensis* (1 case), *L.panamensis*/*L.guyanensis* (1 case), *L. mexicana* complex (1 case). In treated patients, drugs were well tolerated, cure were documented and no relapse have been currently reported (3 years later).

**Conclusions:**

Clinical characteristics, demographic data, and epidemiological features of infection for each case evidence the potential risk related with travel to endemic areas of leishmaniasis.

**Keyworks:**

Cutaneous leishmaniasis, Epidemiology, Imported cases.

## Background

Leishmaniasis is a vector-borne diseases caused by around 20 species of the genus *Leishmania* which are transmitted through the bite of female sandflies to mammalian hosts. Different clinical presentations, including cutaneous (CL), mucocutaneous (MCL), and visceral leishmaniasis (VL), are present in 98 countries worldwide; while 2 million new cases are reported per year [1]. The epidemiology of leishmaniasis is dynamic and the circumstances of transmission are continually changing concerning the environment, demography, human behavior, socioeconomic status, and other factors [2]. In line with this, an increase in cases due to migration, traveling, and military conflicts have been notified around the world in the recent past [3–5].

In particular, the American continent represents a special scenario for the disease due to: (i) a high disease burden, (ii) competent vectors for transmission, (iii) circulation of 20 different species in the geographical area, (iv) up to ten different species within the same territory/country (per example 15 species have been reported in Brazil and 11 in Peru), and (v) at least 26 animal reservoir, human included [6]. The disease is present in 19 countries, with important transmission incidence from Mexico to Argentina with 66,941 cases [1]. Argentina, Brazil, Colombia, Ecuador, Venezuela, Paraguay and Perú report stable transmission [6–8]. However, information about imported cases in non-endemic countries from Latin America is extremely scarce.

In Cuba, leishmaniasis is not endemic [9]; although few imported cases with cutaneous leishmaniasis were diagnosed and treated in the ‘70s and ‘80s of the last decade, which were not reported (Statistical Department of Institute of Tropical Medicine Pedro Kourí). Recently, five out of 16 suspicious imported cases investigated in our laboratory in 10 years (2006–2016) were confirmed and documented as leishmaniasis. The rest of 11 received cases from this serie, had a final diagnosis other than leishmaniasis, including psoriasis, T-cell lymphoma, typhoid fever, leprosy, hyper ascaridiasis, or lympho-monocitary vasculitis [9]. They all shared a common epidemiologic feature since the patients in this study visited different settings where contact with vectors was possible. In general, several factors such as human activities and tourism could increase the risk in the number of *Leishmania* cases. In this sense, a continuous surveillance and international sanitary control is a permanent task nowadays. However, health professionals must be alert and when interrogating the patients take into consideration the possibilities of conditions favoring the spread of this disease.

In particular, during 2017, an increase of suspicious leishmaniasis imported cases arriving in Cuba was observed, which motivate to perform an individual and general analysis. In the present report, the epidemiological contexts of infections, their clinical presentation, the diagnostic methods used, treatment and follow up are described. This will serve not only as an update on the disease in the country context but also as a contribution to the discussion about the influence of human movement in the epidemiology of this parasitic disease.

## Methods

### Study area

The present study took place at the Institute of Tropical Medicine Pedro Kouri (IPK), Havana, Cuba, where five cases of suspicious of leishmaniasis arrived during 2017. The regulations of the Institutional Ethical Committee at IPK (CEI-IPK-8918) concerning the use of human clinical samples for research purposes were respected. All the patients voluntarily participated, signed the agreement through the informed consent for the use of their information, clinical samples and photographs for diagnostic, research, and academic purposes.

### Study subjects and clinical evaluation

Patients (with code: 17–01, 17–02, 17–03, 17–04, and 17–05, in order of arrival date) were received in the IPK out patient clinic as suspicious cases of leishmaniasis from January to December 2017. They all had cutaneous lesions in different locations and variable time of evolution. Personal and general data about each one was obtained during interview or from Hospital’s Clinical Records. All the information concerning the epidemiological conditions pertaining to the probable infection was also collected.

Clinical and dermatological evaluation of each patient and their lesion(s) were made at the Hospital from the IPK by physician experts in parasitology and dermatology, who indicated laboratory analysis. Re-evaluations were routinely performed during their admittance in the hospital, treatment administration and follow-up period.

### Sample collection and diagnostic

Skin biopsies were taken from more than one lesion/patient whenever possible. Scrapings (sterile lancets) and biopsy (disposable punches) were taken from the edge of the lesions according to their location and time of evolution, preferring active lesions. The algorithm followed for diagnosis comprised the use of parasitological and molecular tools to analyze the samples. Firstly, smears were prepared from the lesion’s material, fixed with methanol and stained with Giemsa for detecting intracellular parasites (amastigotes) under light microscopy at 1000x. Five microns thick tissue sections were obtained from paraffin-embedded skin tissues, stained with hematoxilin-eosin and analyzed under light microscopy with at 1000x, except for case 17–05.

### Species identification

A portion of fresh skin tissue from each patient was used for DNA extraction with High Pure PCR Template Preparation Kit (Roche, Germany) following the manufacturer’s instructions. Then, PCR targeting the 18S rRNA (namely PCR-18S) was performed, using the primers and conditions described by Deborggraeve et al. [10] for *Leishmania* genus detection. Afterwards, PCR-F and PCR-N assays, which amplify different fragments of *hsp*70 gene and their corresponding stepwise RFLP’s algorithm were previously described by Montalvo et al. [11] and Fraga et al. [12], to determine the infecting species.

### Treatment

All the patients considered as positive for leishmaniasis that remained in Cuba after diagnosis were treated with conventional drugs. According guidelines exposed by PAHO and evidence-based recommendations [13], the following formulations were selected: Amphotericin B® (deoxycholate 50 mg/ampule; Empresa Laboratorios AICA, La Habana, Cuba), Ampholip® (Amphotericin B Lipid Complex 50 mg/vial; Bharat Serums And Vaccines Limited, Maharashtra, India) and Fluconazol® (150 mg/capsule; Empresa Laboratorios MEDSOL, La Habana, Cuba). The cure was defined if complete reepithelization of the cutaneous tissue was clinically observed without surging of new lesions. On the other hand, therapeutic failure was defined as the presence of active lesions at day 30 / 60 / 90 or 180 after last treatment (time of re-evaluation). Patients were followed up for at least 1 year after the end of the last treatment regimen, including clinical and laboratory evaluations.

## Results

**Fig. 1:**
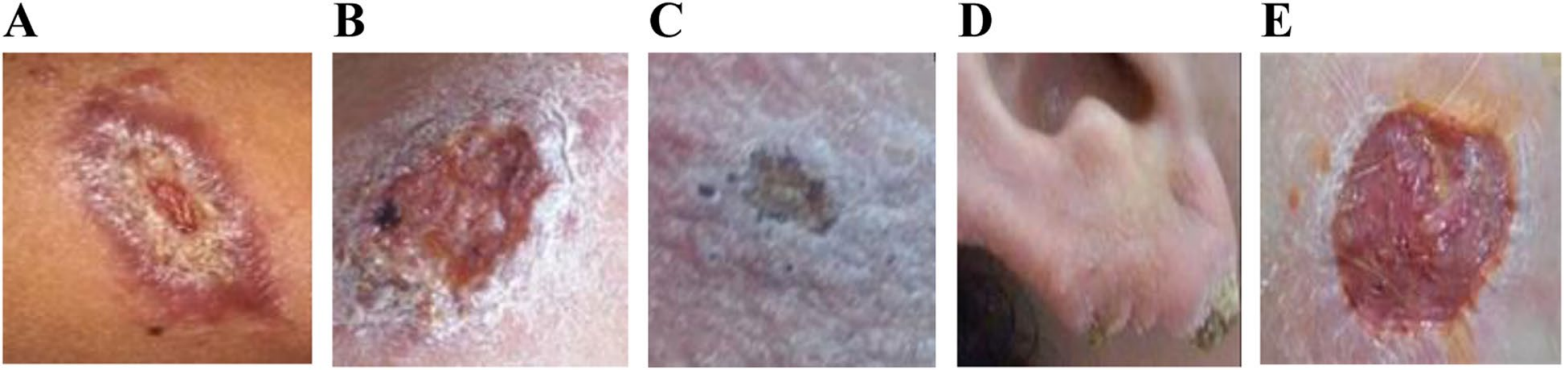
Lesions and clinic description of patients with cutaneous leishmaniasis. IPK, Havana, Cuba, 2017 (Patients appear in chronological order to arrive at IPK). **A** Case 17–01 (A lesion in the external upper part of the nose, boarding with the palpebral area (1 ½ cm diameter). Another one in the left arm (3 ½ cm diameter). Both active, well defined and ulcerated); **B** Case 17–02 (An active lesion in the external side of the left leg (4 cm diameter). Border defined, ulcer and crust, over-infected and purulent.); **C** Case 17–03 (Two lesions ulcerated, crusty, on both sides of the back. Other numerous ones smaller, all around the area and near the shoulder.); **D** Case 17–04 (Several lesions in the border of the right ear. Lesions were crusty and over-infected); **E** Case 17–05 (One lesion in the right leg, ulcerated and not crusty)

*Leishmania* infection was confirmed in all five cases suspicious of having the disease. Figure 1A-E showed the lesion of each patient at arrival time and clinical description, which was consistent with *Leishmania*. No systemic signs of infection nor other symptoms were appreciated and according to the laboratory test performed, all patients were immunocompetent and common laboratory tests within normal ranges.

Epidemiological data of the five patients were summarized in Table 1, which 4/5 (80%) were men and 1/5 (20%) female. Four were Cuban citizens (80%) and the other one (20%) to a British residing in Mexico. In all cases, the epidemiological background supported the possible natural infection, through the bite of *Leishmania* vectors when their natural environment was invaded.

**Table 1:** General epidemiological data of patients with cutaneous leishmaniasis that arrived to IPK, Havana, Cuba, 2017

Case	Sex / Age	Depart	Route
17–01	Female / 49	Havana	Guyana, Brazil, Peru, Ecuador, Colombia (Turbo), Panama (Darien Jungle), Costa Rica, Nicaragua, Honduras, Guatemala, Mexico
17–02	Male / 55	Havana	Guyana, Venezuela, Colombia, Panama, Costa Rica, Nicaragua, Honduras, Guatemala, Mexico
17–03	Male/39	Havana	Guyana, Suriname, Guyana
17–04	Male/27	Havana	Brazil, Peru, Ecuador, Colombia (Turbo), Panama (Darien Jungle)
17–05	Male/43	Mexico	–

The countries comprising all the routes followed by each patient before their arrival in Cuba are mentioned

All the studied cases corresponded to travelers that passed/crossed through several Latin American countries where parasites and vectors are endemic (Table 1). Three of them (cases 17–01, 17–02, and 17–04) followed irregular trails, including long journey jungle walking, no safe ground nor river transportation. On the other hand, the case 17–03 agreed to be hired for working on mines without actual information about living conditions, exposing himself to the forest, and sleeping outdoors barely protected. Finally, the patient represented as 17–05, traveled straight from Mexico to Cuba by airplane. However, he got probably infected during previous stays in rural areas visited for professional purposes, several weeks before the lesion appearance. Although it was impossible to determine the country or place of infection, two possible “hot spots” for transmission were suggested: Turbo (Colombia) and Darien Jungle (Panama), but other ones could be considered as well.

Related to laboratory diagnostic methods, Table 2 showed that direct microscopic observation of amastigotes was possible in cases 17–01, 17–02, and 17–05; while the histological study was positive for *Leishmania* parasite and kinetoplast in all analyzed biopsies (except for case 17–05 that was not studied). PCR-18S and PCR-N were positive in all five of them; whereas PCR-F was only negative in case 17–05, and the amplicons obtained using PCR-F were weak for 17–03. Then, species or species complex were identified in all cases (Table 2). According to the combined result of PCR-F/RFLP and PCR-N/RFLP, in three cases a unique species could be identified: *L. braziliensis* for case 17–01 and *L. panamensis* for cases 17–02 and 17–04. However, in case 17–03, although PCR-F product was restricted with *Bcc*I, expecting to discriminate between parasites belonging to *L. panamensis*/*L. guyanensis*, the obtained pattern was not conclusive. In this sense, bands corresponding to *L. panamensis* (787 bp, 429 bp) and *L. guyanensis* (544 bp) were obtained. Finally, case 17–05 was found to be infected by a species from *L. mexicana* complex, which cannot be discriminated by either of the approaches used.

**Table 2:** Samples and diagnostic results of patients with cutaneous leishmaniasis. IPK, Havana, Cuba, 2017

Case	Samples	Microscopy	Histology	PCR 18S	PCR F	RFLP F	PCRN	RFLPN	Final Diagnosis
17–01	Lancet scrapings and punch biopsy	+	+	+	+	*L.bra*	+	*L.bra*	*L. braziliensis*
17–02	Lancet scrapings and punch biopsy	+	+	+	+	*L. pan*	+	*L. pan* / *L. guy*	*L. panamensis*
17–03	Lancet scrapings and punch biopsy	–	+	+	+ (w)	*L. pan / L. guy*^*a*^	+	*L. pan / L. guy*	*L.pan/L.guy*
17–04	Lancet scrapings	–	+	+	+	*L. pan*	+	*L. pan / L.guy*	*L. panamensis*
17–05	Lancet scraping	+	ND	+	–	ND	+	*L. mex* complex	*L. mexicana* complex

+: Positive result to diagnostic of *Leishmania* spp

-: Negative result to diagnostic of *Leishmania* spp

*ND* Not done

*L. bra*: *Leishmania braziliensis*

*L. pan*: *Leishmania panamensis*

*L. guy*: *Leishmania guyanensis*

*L. mex*: *Leishmania mexicana*

Positive (w): A weak amplicon was obtained

^a^ The pattern obtained corresponded to bands characterizing *L. panamensis* (787 bp, 429 bp) and *L. guyanensis* (544 bp)

Immediately after diagnostis, patients received treatment; except case 17–05 who returned to his home country without a therapeutic regimen. Treatment and follow up of cases 17–01, 17–02, 17–03 and 17–04, are summarized in Table 3. In general, treatment was tolerated, remission of the lesions was observed in patients and no relapse has been currently reported (3 years later).

**Table 3:** Treatment and follow up of patients with leishmaniasis included in this study

Case 17–01 (BW = 65.5 kg): Ampholip® (25 mg/day 1, 50 mg/day 2, and 150 mg/day 3 until 11), after 2 months Fluconazol® (300 mg/day during 30 days) and cutaneous lesion appears resoluted with negative PCR. One year later, appears new mucosal lesions and was treated with Ampholip® (300 mg/day 1 until 7), Fluconazol® (150/day during 37 days), and Ampholip® (300 mg/day during 7 days and 200 mg/day during 6 days). Finally, mucosal resolution was also observed with negative PCR and negative tomography of perinasal sinuses.	
Case 17–02 (BW = 98 kg): Fluconazol® (300 mg/day during 15 days) in combination with Ampholip® (150 mg/days 7, 8, and 14). At this moment, resolution of lesion was observed with negative PCR.	
Case 17–03 (BW = 100 kg): Amphotericin B® (10 mg/day 1, 20 mg/day 2, 30 mg/day 3, 40 mg/days 4, 5 and 6, and 50 mg/day 7), Fluconazol® (150 mg/day during 30 days) and 3 months later, other cycle of Fluconazol® (750 mg/week during 4 weeks). Ten months later, during reconsult patient received Ampholip® (300 mg/day during 5 days and day 14). After that, resolution of lesion was observed with negative PCR.	
Case 17–04 (BW = 75 kg): Amphotericin B® (10 mg/day 1, 20 mg/day 3, 30 mg/days 4 and 5, and 40 mg/day 8). Fluconazol® (150 mg/day during 20 days). Clinical resolution was observed and patient did not return again to reconsult.	
Case 17–05: Did not receive treatment due to the patient returned to origin country after diagnostic.	

## Discussion

CL is recognized as one of the most frequent skin diseases occurring after traveling in endemic areas [14]. The diagnosis may be also a challenge because unusual presentations can occur [15] and parasitological detection, which is often the most available method, usually relies on technical expertise. Taking all this into consideration, travel clinics and referral centers must be prepared to offer not only a prompt but an accurate diagnosis.

All the cases presented here were positive for *Leishmania*. As a laboratory in a non-endemic area, we continue promoting the use of different methods, eluding subjectivism or inexperience, so parasitological and molecular tools are currently utilized during the diagnostic process. It is known that a combination of laboratory methods increased the sensitivity for diagnosis and also provides the possibility to identify the infecting species [4].

Our results indicated that each case had at least one positive parasitological result whereas molecular detection of DNA was possible in all of them, using more than one target (*hsp*70 and *rDNA* genes) which makes the final diagnostic robust. It is not surprising that weak DNA product had been obtained after PCR-F in case 17–03, due to the amplicon’s size. While PCR-N is 593 bp length, PCR-F is 1286 bp, a feature that can affect diagnostic sensitivity, as it has been previously reported by our group [16].

In regards to typification, the species identified corresponded with those reported in the countries where the infected persons stayed, and the results were concordant according to the RFLP scheme used. The results of PCR-F/RFLP-*Bcc*I in case 17–03 are remarkable. According to the *hsp*70 and *hsp*20 sequences analysis, *L. panamensis* and *L. guyanensis* were previously considered as a monophyletic group [17]. Nevertheless, both of them could be distinguished using *Bcc*I as a restriction enzyme for PCR-F product [18], which was validated in the differentiation of isolates and clinical samples from some endemic countries [19, 20]. However, the pattern obtained after PCR-F/RFLP was not unequivocal, as it showed bands expected for both entities: *L. panamensis* and *L. guyanensis*. Considering that *L. panamensis* have not been reported in the territories visited by patient 17–03, where *L. guyanensis, L. braziliensis* and *L. amazonensis* are main species involved in CL [21], one possible explanation could be that this patient suffering of a mixed infection of *L. guyanensis* and other species. Another possibility is that the pattern observed corresponds to a different *L. guyanensis* population, agreeing with the significant genetic diversity associated with this species reported, for example, infecting miners in that country [22, 23]. As the detection of polymorphism within each species varies according to the genetic markers used, it is possible that further studies, using multilocus sequencing, could shed light on this matter.

Concerning treatment, different protocols were used mainly due to availability of drugs (due to supply chain), since leishmaniasis is a non-endemic diseases with sporadic cases. Then, the selected drug was personalized for each case according dosage and duration of therapy, the clinical aspect of the lesions, and the response of each patient. In addition, a strict follow up of clinical parameters were performed in all cases. We acknowledge that international guidelines exist [24]; specially in non-endemic settings as Cuba where the medical expertise treating leishmaniasis is limited. However, the lower accessibility to drugs in the international market impact into the treatment management. In this context, some alternatives of treatment as were presented, such as the use of a lipid complex of amphotericin B produced in India.

Nevertheless, is evident that for CL a systemic treatment and drugs with different effectiveness against *Leishmania* is considered mandatory. In this regard, in the analyzed cases, drugs targeting ergosterol were used, including amphotericin B that binds to membrane sterols, forming complexes that arrange into ion channels and increase membrane permeability [25] or fluconazole that interfere with ergosterol biosynthesis by inhibiting the C-14 demethylation of sterols in *Leishmania* [26]. In addition, the use of lipid complex of amphotericin B (Ampholip®) represents an advantage, such as: (i) deliver the drug on-site, (ii) minimizing the dosage by many folds, and (iii) reducing the side effects related to drug toxicity, which is preferable over using conventional amphotericin B [27].

Althoutgh cure was achieved for all treated patients, response was very different. However, we can not determined the real causes of this due to the influence of different factors; among them: (i) time elapse between infection and treatment start, (ii) severity of disease when treatment started, (iii) clinical characteristics related with single/multiple or nodule/ulcerated lesion, and (iv) immunological status of patients. Nevertheless, longer and complex treatment was administered to *L. braziliensis* (case 17–01). It is known that *L. braziliensis* is the main causal agent of CL and MCL in the Americas [28] with the greatest relative abundance in Colombia and frequently results in therapeutic failure [29]. Recently, a study to determine drug susceptibility profiles of amphotericin B and fluconazole in cultured isolates of Old World and New World *Leishmania* spp., showed reduced susceptibility to drugs against New World species compared with Old World strains. In particular, some clinical isolates of *L. braziliensis* and *L. panamensis* displayed lower susceptibility compared with reference strain [30]. Nevertheless, therapeutic response is highly variable across the American continent, which could relate with virulence or aggressive behaviour of cinculating strain [31]. In addition, probably spontaneous resolution in patients instead of treatment-dependent responsed is not depreciable.

According to World Health Organization (WHO), leishmaniasis remains as a group of diseases without current control measures. Prophylactic vaccines do not exist nor vector control is effective in most of the settings with a predominant peridomiciliary transmission where these parasitoses are endemic [32, 33], which makes harder the task. Besides, it has been recently recognized that “in an interconnected world, change is occurring across social, environmental and climatic scales affecting human, animal and natural systems” [32] and leishmaniasis is not an exception. Among the multiple epidemiological features surrounding the possible occurrence of leishmaniasis, the human movement between low- and high-risk areas is also important, mainly when uncontrolled displacements take place. In this study, 4 out of 5 cases departed from a country where *Leishmania* is not present, to enter a whole region where the disease is highly distributed [1, 6, 34]. Even more, some of the countries with the highest number of CL cases reported in Latin America, such as Brazil, Peru, Colombia, and Panama [6], were intruded on by most of these persons on their route, increasing, in particular, their risk. As it was described for all the cases (except 17–05), the persons infected traveled rural areas unsafely and slept outdoors without protection. All five cases spent partial or complete journeys in the forest or the jungle; without protecting from insect bites capable of transmitting leishmaniasis or another vector-borne disease. Remarkably, the interviews corroborated that none of the travelers knew about the disease nor other probable infections transmitted by vectors, except for dengue. Therefore, unknowingly they disregarded that possibility and displaced under inappropriate protection measures and totally vulnerable to a serie of transmissible diseases. These cases can serve as an example of the serious risks assumed by persons that decide to travel, in particular, by irregular routes, whatever be the reason.

In Cuba, there is currently no evidence about the presence of recognized *Lutzomyia* species that could transmit *Leishmania* parasite [35]. However, considering the ecology of sandflies, it is unlikely that phlebotomus could enter the country with travelers, adapt to new ecology conditions and expand their habitat. Although the possibility of the disease spreading certainly is low, notification of imported cases is necessary in the age of globalization.

A recent retrospective analysis between 2006 and 2016 showed that in 10 years only 5 patients with positive *Leishmania* infection were confirmed out from 16 suspicious cases. Thus, it may cautiously be assumed to our best knowledge that this series of 5 cases during 2017 (1 year), constitute the great majority of imported cases/year diagnosed and treated in Cuba. In several non-endemic countries, the number of cases has increased in the past decade. In particular, some reports from Europe, like Belgium [36], Poland [37], and Sweden [4], are showing an increase in cases imported from America. In parallel, the human movement towards of the island, not only causes an increment in leishmaniasis (as described herein), same challenge has been also evidenced in other non-endemic parasitic diseases such as trypanosomiasis [38] and malaria [39].

## Conclusion

The results presented herein suggest that new efforts should be addressed in terms of educating the general population from non-endemic diseases in relation to the risk of travelling in unsafely conditions into dangerous geographic zones. In addition, it is necessary to strengthen the continuous education to refresh our physicians in the differential diagnosis of skin lessions from traveleres returning from endemic regions of leishmaniasis in the primary healthcare centre. But for international travelers control centers updating of cutaneous and mucocutaneous leishmaniasis should be mandatory.

## Data Availability

Data related with cases and laboratory tests are available from the corresponding author on reasonable request. All relevant data are within the manuscript.

## Abbreviations

CEI-IPK: Institutional Ethical Committee at Institute of Tropical Medicine Pedro Kouri
CL: Cutaneous leishmaniasis
IPK: Institute of Tropical Medicine Pedro Kouri
MCL: Mucocutaneous leishmaniasis
VL: Visceral leishmaniasis
WHO: World Health Organization
